# Crystal structure of *N*,*N*′-bis­(4-methyl­phen­yl)di­thio­oxamide

**DOI:** 10.1107/S2056989014027911

**Published:** 2015-01-03

**Authors:** Antonino Giannetto, Santo Lanza, Giuseppe Bruno, Francesco Nicoló, Hadi Amiri Rudbari

**Affiliations:** a Department of Chemical Sciences, University of Messina, Via F. Stagno d’Alcontres 31, 98166 Messina, Italy; b Department of Chemistry, University of Isfahan, 81746-73441 Isfahan, Iran

**Keywords:** crystal structure, di­thio­oxamide, ethane­dithio­amide, intra­molecular N—H⋯S hydrogen bonds, C—H..π inter­actions

## Abstract

Two half mol­ecules of the title compound, C_16_H_16_N_2_S_2_, are present in the asymmetric unit and both mol­ecules are completed by crystallographic inversion centers at the mid-points of the central C—C bonds: the lengths of these bonds [1.538 (5) and 1.533 (5) Å] indicate negligible electronic delocalization. The *trans*-di­thio­oxamide fragment in each mol­ecule is characterized by a pair of intra­molecular N—H⋯S hydrogen bonds. In the crystal, mol­ecules are linked by weak C—H..π inter­actions, generating a three-dimensional network.

## Related literature   

For the mesogenic properties of related compounds, see: Aversa *et al.* (1997 ▸, 2000 ▸). For the general procedure for the preparation of secondary and tertiary di­thio­oxamides, see: Lanza *et al.* (1993 ▸, 2000 ▸, 2003 ▸); Rosace *et al.* (1993 ▸). For similar crystal structures, see: Shimanouchi & Sasada (1979 ▸).
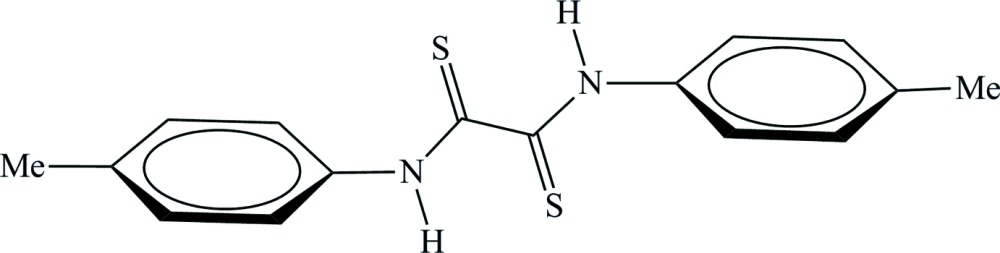


## Experimental   

### Crystal data  

C_16_H_16_N_2_S_2_
*M* *_r_* = 300.43
Monoclinic, 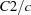

*a* = 33.9423 (7) Å
*b* = 11.3880 (2) Å
*c* = 7.8049 (2) Å
β = 99.439 (1)°
*V* = 2976.02 (11) Å^3^
*Z* = 8
Mo *K*α radiation
μ = 0.35 mm^−1^
*T* = 293 K
0.15 × 0.10 × 0.08 mm

### Data collection  

Bruker APEXII CCD diffractometer
Absorption correction: integration (*SADABS*; Bruker, 2012 ▸) *T*_min_ = 0.657, *T*_max_ = 0.745
45528 measured reflections
2621 independent reflections
1626 reflections with *I* > 2σ(*I*)
*R*_int_ = 0.066

### Refinement  

*R*[*F* ^2^ > 2σ(*F* ^2^)] = 0.055
*wR*(*F* ^2^) = 0.152
*S* = 1.11
2621 reflections
181 parameters
H-atom parameters constrained
Δρ_max_ = 1.03 e Å^−3^
Δρ_min_ = −0.21 e Å^−3^

### 

Data collection: *APEX2* (Bruker, 2012 ▸); cell refinement: *SAINT* (Bruker, 2012 ▸); data reduction: *SAINT*; program(s) used to solve structure: *SHELXS97* (Sheldrick, 2008 ▸); program(s) used to refine structure: *SHELXL97* (Sheldrick, 2008 ▸); molecular graphics: *SHELXTL* (Sheldrick, 2008 ▸); software used to prepare material for publication: *SHELXTL*.

## Supplementary Material

Crystal structure: contains datablock(s) global, I. DOI: 10.1107/S2056989014027911/hb7339sup1.cif

Structure factors: contains datablock(s) I. DOI: 10.1107/S2056989014027911/hb7339Isup2.hkl

Supporting information file. DOI: 10.1107/S2056989014027911/hb7339Isup3.cml

**Fig. 1.**
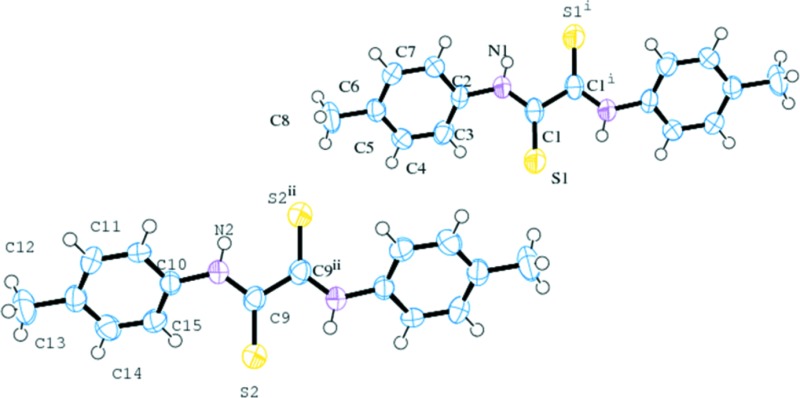
Perspective view of the title molecule with displacement ellipsoids plotted at the 50% probability level, while H atoms are shown as small spheres of arbitrary radius.

**Fig. 2.**
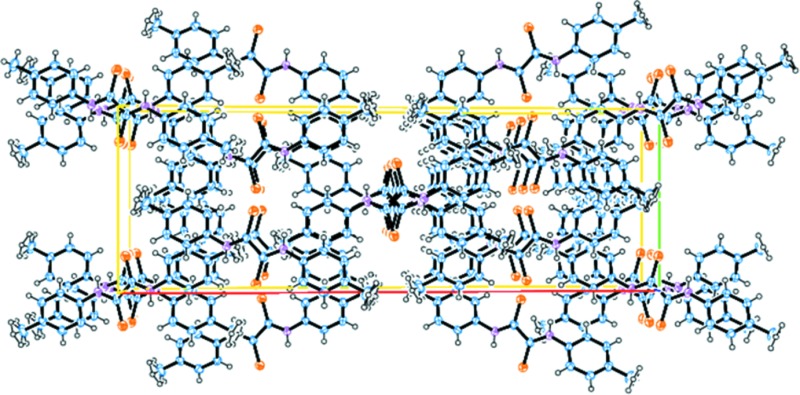
Packing diagram of the title compound viewed along the c axis.

. DOI: 10.1107/S2056989014027911/hb7339fig1.tif
Perspective view of the title mol­ecule with displacement ellipsoids plotted at the 50% probability level, while H atoms are shown as small spheres of arbitrary radius.

c . DOI: 10.1107/S2056989014027911/hb7339fig2.tif
Packing diagram of the title compound viewed along the *c* axis.

**Fig. 3.**
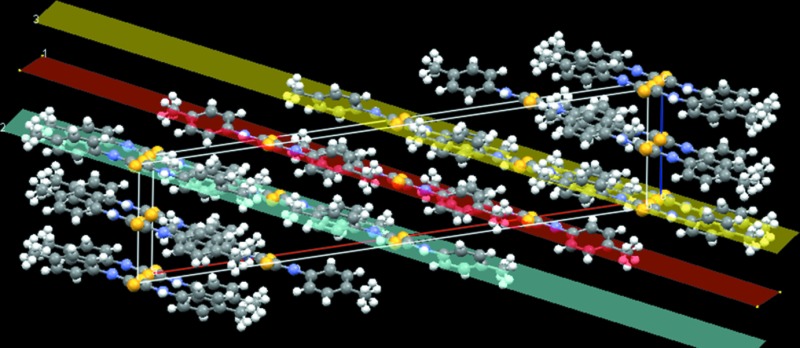
Packing diagram of the title compound viewed normal the b axis and showing molecular arrangement on the (402) plane.

Click here for additional data file.b . DOI: 10.1107/S2056989014027911/hb7339fig3.tif
Packing diagram of the title compound viewed normal the *b* axis and showing mol­ecular arrangement on the (40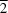) plane.

CCDC reference: 1040609

Additional supporting information:  crystallographic information; 3D view; checkCIF report

## Figures and Tables

**Table 1:** Hydrogen-bond geometry (, ) *Cg*1 and *Cg*2 are the centroids of the C2C7 and C10C15 rings, respectively.

*D*H*A*	*D*H	H*A*	*D* *A*	*D*H*A*
N1H1S1^i^	0.86	2.35	2.904(3)	122
N2H2S2^ii^	0.86	2.35	2.901(3)	122
C7H7*Cg*1^iii^	0.93	2.90	3.587(3)	132
C11H11*Cg*1^iv^	0.93	2.77	3.524(3)	139
C14H14*Cg*2^v^	0.93	2.88	3.654(3)	142

Symmetry codes: (i) 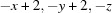; (ii) 
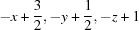
; (iii) 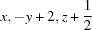; (iv) 
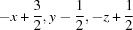
; (v) 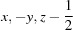.

## supplementary crystallographic information

### S1. Comment

Aryl-substituted secondary dithiooxamides H_2_N_2_C_2_S_2_*R*_2_ (*R*=-C_6_H_4_*X*, C_6_H_3_*X*_2_, *X* =-O-(CH_2_)*_n_*-CH_3_, 7 <n > 11) have been exploited to prepare platinum(II) complexes which exhibit mesogenic properties (Aversa, *et al.* 1997; Aversa, *et al.* 2000). The title compound has been synthesized for a better understanding of the reactivity of the said mesogenic complexes, in the aim to avoid contingent steric hindrance of long chain substituents and the influence on the acid base equilibria of ether moieties. In fact, both the oxygen lone pairs in ether moieties and the exceedingly long alkyl chains of *X* substituents prevents the formation of ion pairs in the first step of the reaction of secondary dithioxamides with *cis*-Pt(Me_2_SO)_2_Cl_2_ (Lanza *et al.*, 1993). A detailed analysis of the bond distances reveals a strong double-bond character for both C—S and C—N [1.662 (3) Å and 1.318 (4) Å, respectively], confirming that the important electronic π-delocalization of the N—C—S system does not affect the central C—C bond. In the title compound the central C—C bond distances are 1.538 (5) Å and 1.533 (5) Å respectively for C1—C1' and C9—C9'. Going from a secondary dithiooxamide to a tertiary one we observe a large changing in structural parameters:essentially in the planarity loss of the central fragment and to the significant shortening of central C—C bond. The *p*-tolyl groups are rotated by -36.4° (5) and -35.4° (5) with respect to the central DTO fragments.

### S2. Experimental

The title compound was obtained from *para*-toluidine according to a described two-step strategy based on primary amine reaction with oxalyl chloride followed by P_2_S_5_ treatment. ^1^H NMR: δ 12.33 (bs, NH), 7.92 (d, J*_ortho_* 8.4 Hz, H_2,6_), 7.28 (d, H_3,5_), 2.40 (s, Me). ^13^CNMR: &delta; 180.56 (CS), 137,75 (C_4_), 135.80 (C_1_), 129.64 (C_3,5_), 122.17 (C_2,6_), 21.27 (Me)

### S3. Refinement

H atoms on methyl groups were included in the refinement as idealized disordered in two positions, others H atoms were included in the refinement among the riding model method with the *X*—H bond geometry and the H isotropic displacement parameter depending on the parent atom *X*.

### Crystal data

**Table:**

C_16_H_16_N_2_S_2_	*F*(000) = 1264
*M**_r_* = 300.43	*D*_x_ = 1.341 Mg m^−^^3^
Monoclinic, *C*2/*c*	Mo *K*α radiation, λ = 0.71073 Å
Hall symbol: -C 2yc	Cell parameters from 141 reflections
*a* = 33.9423 (7) Å	θ = 4.3–22.0°
*b* = 11.3880 (2) Å	µ = 0.35 mm^−^^1^
*c* = 7.8049 (2) Å	*T* = 293 K
β = 99.439 (1)°	Prismatic, orange
*V* = 2976.02 (11) Å^3^	0.15 × 0.10 × 0.08 mm
*Z* = 8	

### Data collection

**Table:**

Bruker APEXII CCD diffractometer	2621 independent reflections
Radiation source: fine-focus sealed tube	1626 reflections with *I* > 2σ(*I*)
Graphite monochromator	*R*_int_ = 0.066
φ and ω scans	θ_max_ = 25.0°, θ_min_ = 1.2°
Absorption correction: integration (*SADABS*; Bruker, 2012)	*h* = −40→40
*T*_min_ = 0.657, *T*_max_ = 0.745	*k* = −13→13
45528 measured reflections	*l* = −9→9

### Refinement

**Table:**

Refinement on *F*^2^	0 restraints
Least-squares matrix: full	Hydrogen site location: inferred from neighbouring sites
*R*[*F*^2^ > 2σ(*F*^2^)] = 0.055	H-atom parameters constrained
*wR*(*F*^2^) = 0.152	*w* = 1/[σ^2^(*F*_o_^2^) + (0.078*P*)^2^ + 1.595*P*] where *P* = (*F*_o_^2^ + 2*F*_c_^2^)/3
*S* = 1.11	(Δ/σ)_max_ = 0.001
2621 reflections	Δρ_max_ = 1.03 e Å^−^^3^
181 parameters	Δρ_min_ = −0.20 e Å^−^^3^

### Special details

**Table:**

Geometry. All e.s.d.'s (except the e.s.d. in the dihedral angle between two l.s. planes) are estimated using the full covariance matrix. The cell e.s.d.'s are taken into account individually in the estimation of e.s.d.'s in distances, angles and torsion angles; correlations between e.s.d.'s in cell parameters are only used when they are defined by crystal symmetry. An approximate (isotropic) treatment of cell e.s.d.'s is used for estimating e.s.d.'s involving l.s. planes.

### Fractional atomic coordinates and isotropic or equivalent isotropic displacement parameters (Å^2^)

**Table:**

	*x*	*y*	*z*	*U*_iso_*/*U*_eq_	Occ. (<1)
S1	0.99746 (3)	0.81087 (8)	0.00428 (12)	0.0493 (3)	
N1	0.94997 (8)	0.9912 (2)	−0.1064 (3)	0.0362 (7)	
H1	0.9486	1.0665	−0.115	0.043*	
C1	0.98496 (9)	0.9507 (3)	−0.0303 (4)	0.0325 (7)	
C2	0.91413 (10)	0.9314 (3)	−0.1764 (4)	0.0319 (8)	
C3	0.91366 (10)	0.8248 (3)	−0.2619 (4)	0.0376 (8)	
H3	0.9375	0.7876	−0.2728	0.045*	
C4	0.87745 (10)	0.7739 (3)	−0.3311 (4)	0.0341 (8)	
H4	0.8773	0.7022	−0.3883	0.041*	
C5	0.84155 (10)	0.8273 (3)	−0.3169 (4)	0.0329 (8)	
C6	0.84256 (10)	0.9343 (3)	−0.2318 (4)	0.0347 (8)	
H6	0.8187	0.9716	−0.221	0.042*	
C7	0.87853 (10)	0.9868 (3)	−0.1626 (4)	0.0335 (8)	
H7	0.8787	1.0591	−0.107	0.04*	
C8	0.80238 (11)	0.7709 (4)	−0.3933 (5)	0.0527 (11)	
H8A	0.7807	0.8197	−0.371	0.079*	0.5
H8B	0.8004	0.6952	−0.3411	0.079*	0.5
H8C	0.8011	0.7618	−0.5164	0.079*	0.5
H8D	0.8074	0.6981	−0.448	0.079*	0.5
H8E	0.7878	0.8226	−0.4779	0.079*	0.5
H8F	0.787	0.756	−0.3026	0.079*	0.5
S2	0.74804 (3)	0.06064 (8)	0.49865 (14)	0.0539 (3)	
C9	0.73490 (9)	0.2010 (3)	0.4708 (4)	0.0317 (7)	
C10	0.66362 (9)	0.1808 (3)	0.3324 (4)	0.0298 (7)	
C11	0.62785 (10)	0.2328 (3)	0.3544 (4)	0.0298 (7)	
H11	0.628	0.3029	0.4157	0.036*	
C12	0.59213 (10)	0.1807 (3)	0.2856 (4)	0.0350 (8)	
H12	0.5683	0.2158	0.3016	0.042*	
C13	0.59114 (10)	0.0767 (3)	0.1928 (4)	0.0349 (8)	
C14	0.62709 (11)	0.0269 (3)	0.1714 (4)	0.0383 (9)	
H14	0.6269	−0.0427	0.1088	0.046*	
C15	0.66335 (10)	0.0771 (3)	0.2398 (4)	0.0351 (8)	
H15	0.6872	0.0417	0.2239	0.042*	
C16	0.55213 (12)	0.0194 (4)	0.1170 (5)	0.0542 (11)	
H16A	0.5304	0.0656	0.1446	0.081*	0.5
H16B	0.551	−0.0579	0.1649	0.081*	0.5
H16C	0.5502	0.014	−0.0069	0.081*	0.5
H16D	0.5573	−0.0512	0.0572	0.081*	0.5
H16E	0.5367	0.0724	0.0368	0.081*	0.5
H16F	0.5375	0.0005	0.2086	0.081*	0.5
N2	0.69939 (8)	0.2412 (2)	0.4032 (3)	0.0345 (7)	
H2	0.6974	0.3165	0.4014	0.041*	

### Atomic displacement parameters (Å^2^)

**Table:**

	*U*^11^	*U*^22^	*U*^33^	*U*^12^	*U*^13^	*U*^23^
S1	0.0357 (5)	0.0427 (6)	0.0649 (6)	−0.0054 (4)	−0.0051 (4)	0.0079 (4)
N1	0.0268 (17)	0.0327 (16)	0.0462 (16)	−0.0045 (13)	−0.0025 (13)	0.0003 (12)
C1	0.0267 (16)	0.0429 (18)	0.0283 (15)	−0.0072 (15)	0.0052 (12)	0.0001 (14)
C2	0.0230 (17)	0.039 (2)	0.0318 (16)	−0.0040 (16)	−0.0003 (13)	0.0034 (15)
C3	0.0277 (18)	0.045 (2)	0.0396 (18)	0.0023 (17)	0.0048 (14)	−0.0032 (16)
C4	0.037 (2)	0.0299 (19)	0.0350 (17)	−0.0013 (16)	0.0028 (15)	−0.0056 (14)
C5	0.0317 (19)	0.035 (2)	0.0302 (16)	−0.0052 (17)	0.0000 (14)	0.0018 (15)
C6	0.0272 (18)	0.037 (2)	0.0382 (17)	0.0032 (16)	0.0015 (14)	0.0008 (15)
C7	0.0325 (19)	0.0290 (18)	0.0372 (17)	−0.0011 (15)	0.0006 (14)	0.0003 (14)
C8	0.034 (2)	0.063 (3)	0.059 (2)	−0.017 (2)	0.0014 (18)	−0.013 (2)
S2	0.0350 (5)	0.0349 (5)	0.0861 (7)	−0.0018 (4)	−0.0067 (5)	0.0092 (5)
C9	0.0255 (16)	0.0361 (17)	0.0341 (16)	−0.0004 (14)	0.0063 (13)	0.0050 (13)
C10	0.0256 (17)	0.0305 (19)	0.0322 (15)	−0.0034 (15)	0.0011 (13)	0.0031 (14)
C11	0.0300 (18)	0.0228 (17)	0.0361 (16)	0.0048 (14)	0.0041 (14)	−0.0014 (13)
C12	0.0229 (17)	0.044 (2)	0.0376 (17)	0.0019 (16)	0.0033 (14)	−0.0002 (15)
C13	0.0293 (19)	0.040 (2)	0.0335 (17)	−0.0041 (17)	0.0001 (14)	0.0033 (16)
C14	0.042 (2)	0.035 (2)	0.0369 (17)	−0.0029 (17)	0.0026 (16)	−0.0066 (15)
C15	0.0305 (18)	0.034 (2)	0.0415 (18)	0.0048 (16)	0.0071 (14)	−0.0055 (15)
C16	0.039 (2)	0.061 (3)	0.060 (2)	−0.017 (2)	0.0008 (19)	−0.015 (2)
N2	0.0249 (16)	0.0296 (16)	0.0475 (16)	−0.0021 (12)	0.0017 (13)	−0.0009 (12)

### Geometric parameters (Å, º)

**Table:**

S1—C1	1.659 (3)	S2—C9	1.664 (3)
N1—C1	1.321 (4)	C9—N2	1.316 (4)
N1—C2	1.423 (4)	C9—C9^ii^	1.534 (6)
N1—H1	0.86	C10—C15	1.383 (5)
C1—C1^i^	1.538 (6)	C10—C11	1.387 (4)
C2—C7	1.383 (5)	C10—N2	1.426 (4)
C2—C3	1.384 (5)	C11—C12	1.378 (4)
C3—C4	1.386 (5)	C11—H11	0.93
C3—H3	0.93	C12—C13	1.385 (5)
C4—C5	1.383 (5)	C12—H12	0.93
C4—H4	0.93	C13—C14	1.381 (5)
C5—C6	1.386 (5)	C13—C16	1.508 (5)
C5—C8	1.508 (5)	C14—C15	1.383 (5)
C6—C7	1.387 (4)	C14—H14	0.93
C6—H6	0.93	C15—H15	0.93
C7—H7	0.93	C16—H16A	0.96
C8—H8A	0.96	C16—H16B	0.96
C8—H8B	0.96	C16—H16C	0.96
C8—H8C	0.96	C16—H16D	0.96
C8—H8D	0.96	C16—H16E	0.96
C8—H8E	0.96	C16—H16F	0.96
C8—H8F	0.96	N2—H2	0.86
			
C1—N1—C2	130.9 (3)	N2—C9—C9^ii^	112.9 (3)
C1—N1—H1	114.6	N2—C9—S2	126.5 (2)
C2—N1—H1	114.6	C9^ii^—C9—S2	120.6 (3)
N1—C1—C1^i^	112.7 (3)	C15—C10—C11	119.9 (3)
N1—C1—S1	126.6 (2)	C15—C10—N2	123.1 (3)
C1^i^—C1—S1	120.7 (3)	C11—C10—N2	116.9 (3)
C7—C2—C3	119.8 (3)	C12—C11—C10	120.0 (3)
C7—C2—N1	117.0 (3)	C12—C11—H11	120
C3—C2—N1	123.1 (3)	C10—C11—H11	120
C2—C3—C4	119.6 (3)	C11—C12—C13	121.1 (3)
C2—C3—H3	120.2	C11—C12—H12	119.4
C4—C3—H3	120.2	C13—C12—H12	119.4
C5—C4—C3	121.4 (3)	C14—C13—C12	117.9 (3)
C5—C4—H4	119.3	C14—C13—C16	120.8 (3)
C3—C4—H4	119.3	C12—C13—C16	121.3 (3)
C4—C5—C6	118.2 (3)	C13—C14—C15	122.1 (3)
C4—C5—C8	120.8 (3)	C13—C14—H14	118.9
C6—C5—C8	121.0 (3)	C15—C14—H14	118.9
C5—C6—C7	121.1 (3)	C14—C15—C10	119.0 (3)
C5—C6—H6	119.5	C14—C15—H15	120.5
C7—C6—H6	119.5	C10—C15—H15	120.5
C2—C7—C6	119.9 (3)	C13—C16—H16A	109.5
C2—C7—H7	120.1	C13—C16—H16B	109.5
C6—C7—H7	120.1	H16A—C16—H16B	109.5
C5—C8—H8A	109.5	C13—C16—H16C	109.5
C5—C8—H8B	109.5	H16A—C16—H16C	109.5
H8A—C8—H8B	109.5	H16B—C16—H16C	109.5
C5—C8—H8C	109.5	C13—C16—H16D	109.5
H8A—C8—H8C	109.5	H16A—C16—H16D	141.1
H8B—C8—H8C	109.5	H16B—C16—H16D	56.3
C5—C8—H8D	109.5	H16C—C16—H16D	56.3
H8A—C8—H8D	141.1	C13—C16—H16E	109.5
H8B—C8—H8D	56.3	H16A—C16—H16E	56.3
H8C—C8—H8D	56.3	H16B—C16—H16E	141.1
C5—C8—H8E	109.5	H16C—C16—H16E	56.3
H8A—C8—H8E	56.3	H16D—C16—H16E	109.5
H8B—C8—H8E	141.1	C13—C16—H16F	109.5
H8C—C8—H8E	56.3	H16A—C16—H16F	56.3
H8D—C8—H8E	109.5	H16B—C16—H16F	56.3
C5—C8—H8F	109.5	H16C—C16—H16F	141.1
H8A—C8—H8F	56.3	H16D—C16—H16F	109.5
H8B—C8—H8F	56.3	H16E—C16—H16F	109.5
H8C—C8—H8F	141.1	C9—N2—C10	130.8 (3)
H8D—C8—H8F	109.5	C9—N2—H2	114.6
H8E—C8—H8F	109.5	C10—N2—H2	114.6

Symmetry codes: (i) −*x*+2, −*y*+2, −*z*; (ii) −*x*+3/2, −*y*+1/2, −*z*+1.

### Hydrogen-bond geometry (Å, º)

Cg1 and Cg2 are the centroids of the C2–C7 and C10–C15 rings, respectively.

**Table:**

*D*—H···*A*	*D*—H	H···*A*	*D*···*A*	*D*—H···*A*
N1—H1···S1^i^	0.86	2.35	2.904 (3)	122
N2—H2···S2^ii^	0.86	2.35	2.901 (3)	122
C7—H7···*Cg*1^iii^	0.93	2.90	3.587 (3)	132
C11—H11···*Cg*1^iv^	0.93	2.77	3.524 (3)	139
C14—H14···*Cg*2^v^	0.93	2.88	3.654 (3)	142

Symmetry codes: (i) −*x*+2, −*y*+2, −*z*; (ii) −*x*+3/2, −*y*+1/2, −*z*+1; (iii) *x*, −*y*+2, *z*+1/2; (iv) −*x*+3/2, *y*−1/2, −*z*+1/2; (v) *x*, −*y*, *z*−1/2.
