# Supplementary figures and images for: Crystal structure of N,N′-bis­(4-methyl­phen­yl)di­thio­oxamide
Source: Acta Crystallogr E Crystallogr Commun. 2015 Jan 3;71(Pt 2):o67. doi: 10.1107/S2056989014027911 (PMC4384591; doi:10.1107/S2056989014027911)

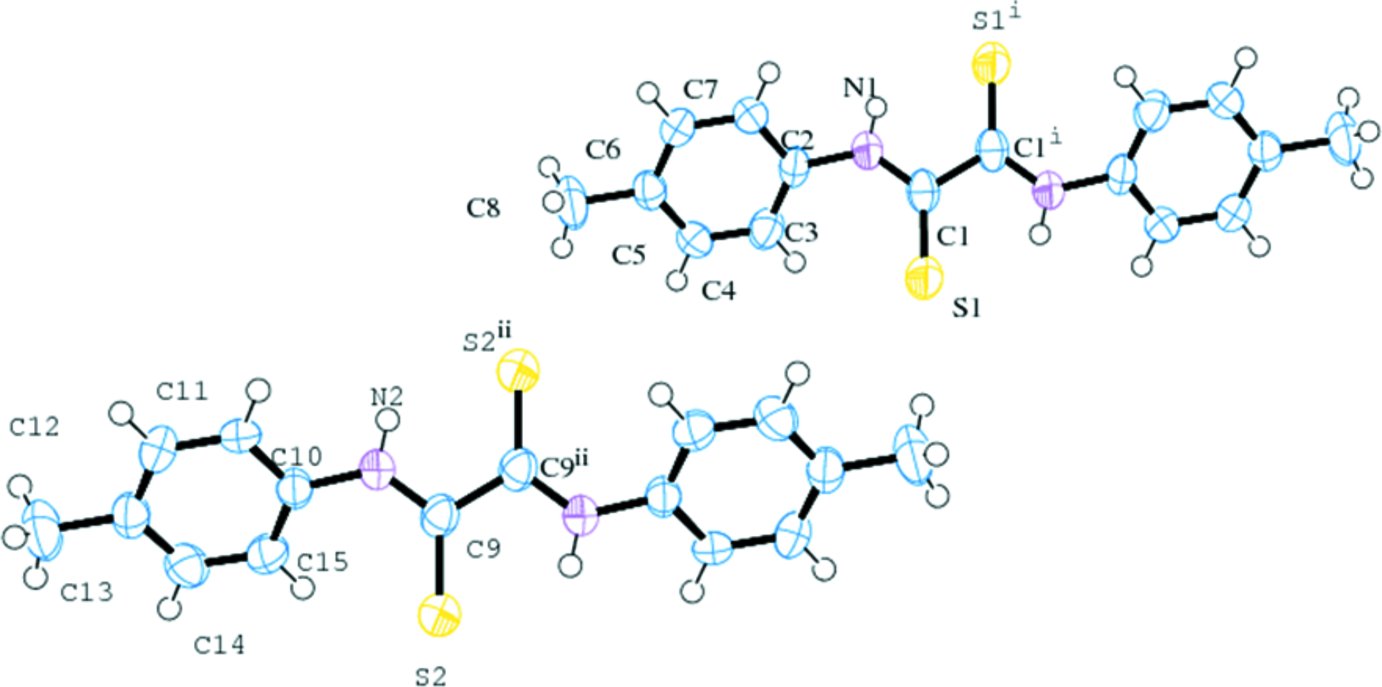

Supplement: Supplementary file 4 [file e-71-00o67-fig1.tif]

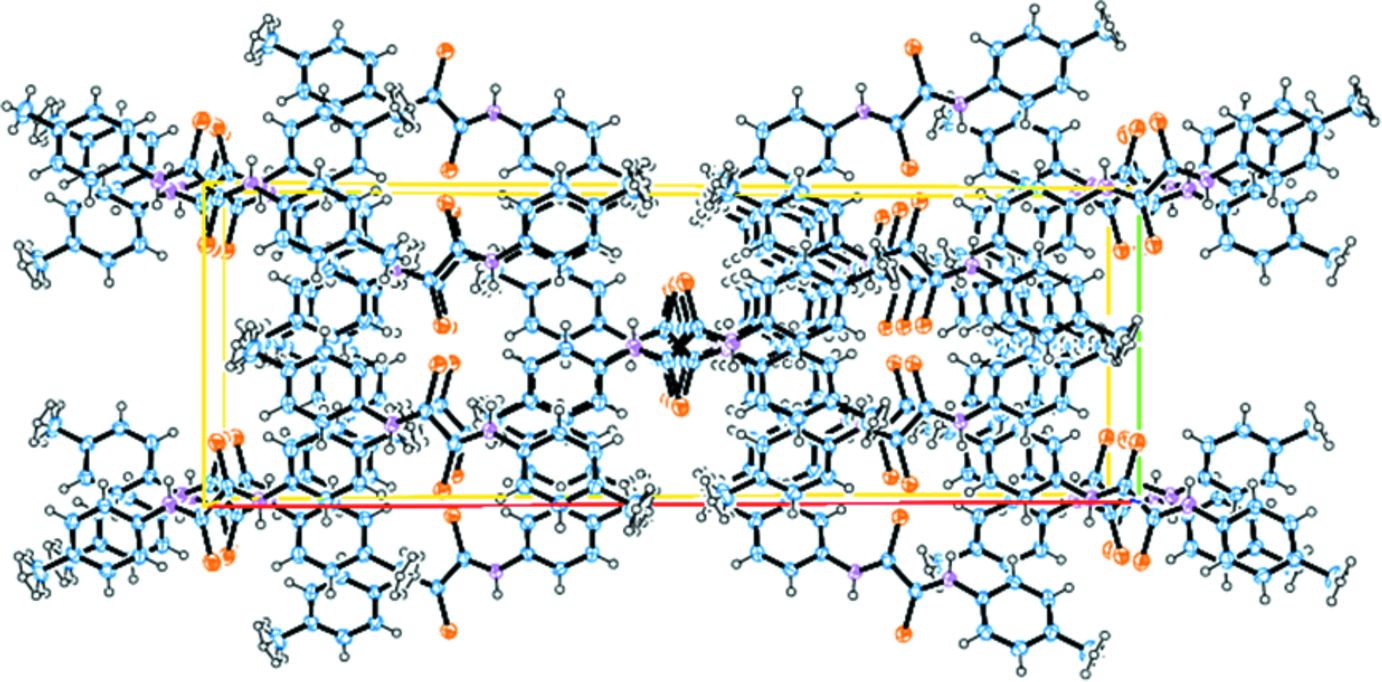

Supplement: Supplementary file 5 [file e-71-00o67-fig2.tif]

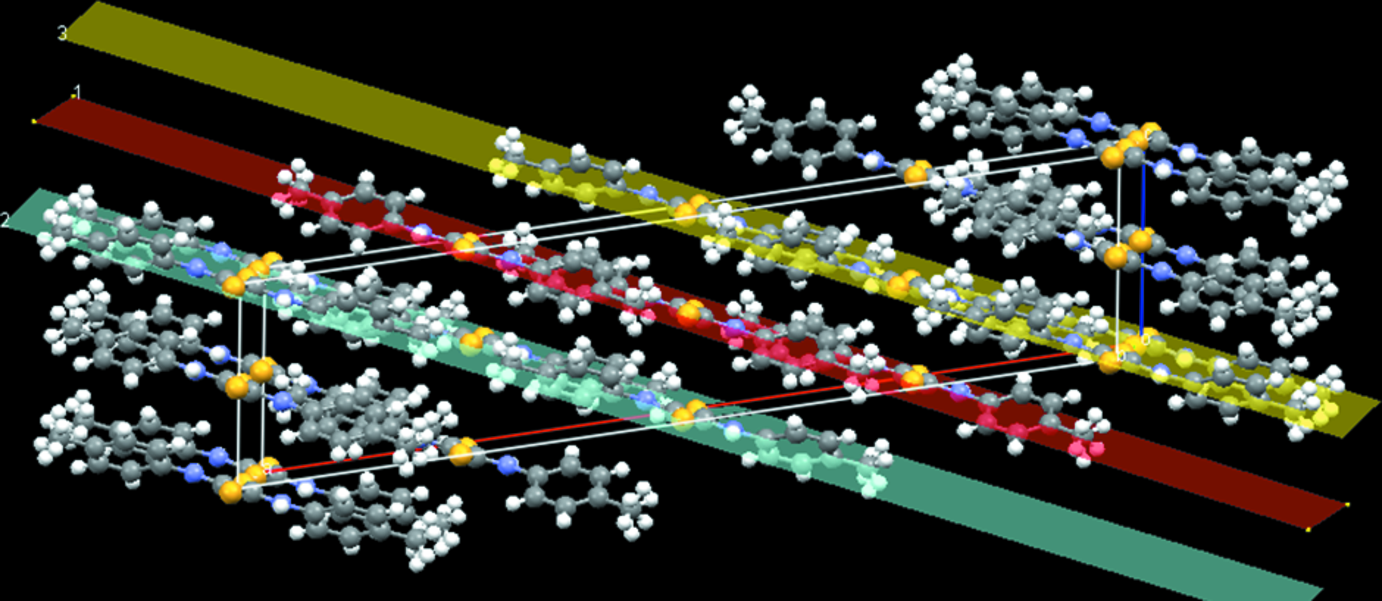

Supplement: Supplementary file 6 [file e-71-00o67-fig3.tif]
